# Systemic therapy in pancreatic ductal adenocarcinomas (PDACs)—basis and current status

**DOI:** 10.3332/ecancer.2022.1367

**Published:** 2022-03-24

**Authors:** Anant Ramaswamy, Sujay Srinivas, Vikram Chaudhari, Prabhat Bhargava, Manish Bhandare, Shailesh V Shrikhande, Vikas Ostwal

**Affiliations:** 1 Department of Medical Oncology, Tata Memorial Hospital, Dr E Borges Road, Parel, Mumbai 400012, India; 2 GI and HPB Services, Tata Memorial Hospital, Homi Bhabha National Institute, Dr E Borges Road, Parel, Mumbai 400012, India

**Keywords:** pancreatic cancer, systemic therapy, India, review, chemotherapy

## Abstract

A major shift in the approach to the management of pancreatic ductal adenocarcinomas (PDACs) has been the recognition of the systemic nature of the disease even in clinically and radiologically limited disease stages. The recalcitrant nature of PDAC is intrinsically related to the lack of therapeutic targets and dense surrounding stroma that limits the activity of currently available chemotherapeutic options. However, research is increasingly focusing on intensifying systemic management options in PDAC, resulting in gradual improvements in survival. Currently effective chemotherapeutic regimens like modified 5-fluorouracil-leucovorin-irinotecan-oxaliplatin and gemcitabine-nab-paclitaxel have improved outcomes in resectable and advanced PDAC. An increasing use of these regimens has also resulted in greater conversion of borderline resectable and locally advanced cancers to resection, though the most effective approach in this subgroup is yet to be identified. The current review presents an outline of the basic systemic nature of PDAC and current options of systemic therapy, predominantly chemotherapy .

## Introduction

Pancreatic ductal adenocarcinomas (PDACs) are the twelfth most common cancer across the world in terms of incidence, but cause a disproportionate number of cancer related death in terms of proportions as per data from GLOBOCAN 2020 [1]. While there appears to be a greater incidence of PDAC in Europe and North America, there is a trend towards an increasing incidence across geographic regions [2]. A similar pattern has been noted in mortality rates, though 5-year survival rates across stages of PDAC have marginally improved from 6% to 9% over the course of the last two decades [1]. A corollary to this finding is that a majority of patients are diagnosed with PDAC in advanced or metastatic stage, where overall survivals (OSs) are in the region of approximately 9–12 months [3].

A major shift in the approach to the management of PDAC has been the recognition of the systemic nature of the disease even in clinically and radiologically limited disease stages [4]. While high-quality surgery forms an important backbone of the management of limited stage PDAC, current research is increasingly focusing on adequate control of systemic PDAC combined with local methods of disease control. This has resulted in gradual improvements in survival for patients with PDAC, though the quantum of improvement lags behind that seen in a number of other malignancies. The basis for systemic management of PDAC remains multi-agent chemotherapy, with options like immunotherapy and vaccine approaches yet to bear fruit in the treatment of PDAC [5].

### Molecular landscape of PDAC

Genomic studies and analyses of copy number variations have identified four major mutations in the genomic landscape that drive the oncogenic process of PDAC – activating mutations causing telomeric shortening in KRAS (≥90%, near ubiquitous in PDAC), inactivating mutations in TP53 (approximately 74%), SMAD4 (approximately 31%) and CDKN2A (approximately 35%). Other less common alterations include changes in KDM6A and PREX2, as well as certain hereditary pathogenic mutations in BRCA, BRCA2 and PALB2.

Mutations in KRAS occur early in the pathogenesis of PDAC, while SMAD4 mutations tend to occur late and facilitate increased aggressiveness and progression of PDAC [6]. While mutations in KRAS and TP53 and abnormal CDKN2A have known to correlate with poorer prognosis, there have been disappointing results when attempts to target these genes have been made, e.g. the failure of farnesyl-transferase inhibitors in targeting KRAS [7, 8]. This lack of useful targets has resulted in shifting focus from genomics to transcriptomics.

While individual mutations give information of drivers of PDAC, the lack of firm clinical correlates has resulted in numerous attempts to evaluate the transcriptomic data analysis with clinical features and outcomes [9]. In as early as 2011, Collisson *et al* [10] used hybridisation array-based mRNA expression on only the epithelial component of patient samples with untreated resected PDAC to identify three subtypes: Classical, Quasi-mesenchymal (QM-PDA) and Exocrine-like. Along with correlation with specific genomic and pathological features, the QM-PDA cohort also had decreased survival compared to the other subtypes [10]. Several other classifications have emerged over the last decade, with a recent one by Puleo *et al* [11] subtyping 309 cases of PDAC into basal like, stroma activated, desmoplastic, pure classical and immune classical subtypes. This built upon a previous study classifying PDAC into basal and classical subtypes, while additionally identifying additional sub-classifications. Besides being identified with particular molecular signatures, the subtypes also showed different survival outcomes: the basal-like subtype had the worst outcome (median OS (mOS) – 10.3 months), whereas the pure classical and immune classical subtypes showed good prognosis (mOS values of 43.1 and 37.4 months, respectively). The stroma activated subtype was associated with a poor prognosis, but better as compared to the pure basal-like subtype (mOS – 20.2 months). An important aspect of this classification was its recognition and inclusion of the pancreatic stroma in determining PDAC biology and outcomes [11].

Increasing evidence also suggests that available transcriptomic classifications have firm translational correlates in terms of predicting responses to available systemic chemotherapeutic options [12]. Early results from the COMPASS study, which entailed performing whole genome sequencing and RNA sequencing in patients with advanced PDAC being treated with 5-fluorouracil-leucovorin-irinotecan-oxaliplatin (FOLFIRINOX) or gemcitabine with nab-paclitaxel (GN), divided patients into ‘basal’ and ‘classical’ subtypes as is commonly used in PDAC. They clearly showed that radiological responses as well as survivals with first-line chemotherapy were markedly inferior in the basal subtypes as compared to the classical subtypes [13].

### Why is PDAC difficult to treat?

PDACs are notoriously difficult to treat malignancies, with limited increments in survival over the last two decades. Major reasons for this recalcitrant nature of PDAC are related to the inherent biology of the cancer. While a complete analysis of pancreatic tumour biology is beyond the scope of this review, some of the important aspects as to why the PDACs are resistant to treatment are discussed.

### Systemic nature of PDAC

Elegant mouse models have shown that pancreatic cancer progression is a model in which the seeding of distant organs occurs before, and in parallel to, tumour formation at the primary site [4]. Most patients with pancreatic cancer have metastatic disease at the time of diagnosis, even if not radiologically evident. Epithelial mesenchymal transition (EMT) is considered as one of the key features of metastases acquired by epithelial cells wherein, they lose their epithelial cell like properties and gain invasive properties and stem cell-like features, allowing for metastases. In their mouse model studies using Cre-lox-based mouse model of PDACs, Rhim *et al* [14] have clearly shown that EMT along with bloodstream entry of transformed cells and seeding of distant organs occurs at a stage of PDAC progression previously thought to be preinvasive based on standard histological examination. Even when tumours were at the precancerous stage (pancreatic intraepithelial neoplasia), they were associated with EMT, suggestive of metastatic potential [14].

### Tumour microenvironment (TME) of PDAC

Besides various genomic alterations seen in PDAC, ongoing research has increasingly identified the effect of the tumour microenvironment (TME) on the nature of PDAC. The neoplastic cells in PDAC comprise a minority of the entire tumour tissue, with the vast majority of tumour tissue being contributed by the TME, predominantly comprising dense desmoplastic stroma. The effect of stroma on tumour genotype and phenotype has been captured in the molecular classifications of PDAC as previously mentioned, thereby reiterating the footprint of the stroma on PDAC biology [15].

There are three fundamental components of the pancreatic stroma – extracellular matrix (ECM), vasculature and cancer associated fibroblasts (CAFs). Desmoplasia is a fibrotic reaction caused by an excess of fibroblasts and the deposition of ECM, and this is a hallmark of PDAC. High stromal concentrations of hyaluronic acid have directly correlated with inferior survivals in PDAC. Besides direct correlation with outcomes, ECM purportedly acts as a barrier to chemotherapy. Studies in genetically engineered mouse models have shown that tumour stroma remodelling via inhibition of small molecules such as STAT3 may improve the efficacy of standard gemcitabine chemotherapy and improve outcomes. The ECM also acts as a barrier to the delivery of chemotherapeutic agents by possibly mediating increased interstitial fluid pressure within the tumour, thereby impairing vascular function and tumour permeability [16] . Available evidence suggests that CAFs are active players during the process of cancer initiation, progression and metastasis. Besides being part of bi-directional signalling between neoplastic cells and themselves in terms of oncogenesis, a subset of CAFs called inflammatory CAF may be pro-tumorigenic themselves. IL-6, secreted by CAFs, may also contribute to immune evasion in PDAC. Numerous therapeutic targets against CAFs or CAF receptors in PDAC are under development with some of the more prominent ones under evaluation being insulin-like growth factor 1 (IGF1) receptor, Vitamin D receptor and inhibition of β-catenin nuclear localisation and invasion of cancer cells by all-trans retinoic acid [17].

### Current concepts in management of advanced PDAC

#### Treatment options in advanced PDAC

The treatment of PDACs is based on their initial presentation in terms of resectability status, with a majority of the patients having locally advanced unresectable (LAPC) or metastatic cancers with only 10%–20% having clearly resectable disease at initial presentation [18, 19] (Table 1). This implies that a majority of treatment regimens have initially been evaluated in the advanced setting, before being used in localised PDACs. Systemic chemotherapy in the form of palliative chemotherapy can improve disease related symptoms and quality of life in metastatic pancreatic cancer; while those with LAPC may be initially treated with neoadjuvant intent therapy. Multidisciplinary joint clinic decisions have become the standard for charting out the treatment plan for patients with advanced PDAC.

There have been a small number of validated regimens for the first-line treatment of advanced PDAC. The current first-line regimen has evolved over the years from bolus 5-fluorouracil (5-FU) to gemcitabine to the current era of FOLFIRINOX and GN. Table 2 summarises the major studies evaluating first-line therapy in advanced PDAC. In routine current practice, the FOLFIRINOX regimen is recommended as a standard first-line choice for advanced pancreatic cancer by the National Comprehensive Cancer Network (NCCN), the American Society of Clinical Oncology and the European Society for Medical Oncology (ESMO) for patients with an ECOG performance status score of 0 or 1 and a favourable comorbidity profile [18, 20, 21]. This is based on the results of the pivotal PRODIGE 4-ACCORD11 trial which compared FOLFIRINOX to gemcitabine in metastatic pancreatic cancer patients below 75 years of age. OS was significantly increased, with median survival of 11.1 months for FOLFIRINOX and 6.8 months for the gemcitabine regimen (Hazard ratio (HR) 0.57; 95% confidence interval (CI), 0.45–0.73; *p* < 0.001) [22].

The MPACT trial reported that a combination of GN was superior to gemcitabine alone as a first-line regimen for the treatment of patients with metastatic PDAC. Of 861 patients with an ECOG performance status score of 0–2 in this study, the mOS was 8.5 months in the nab-paclitaxel arm and 6.7 months in the gemcitabine arm (HR, 0.72; 95% CI, 0.62–0.83; *p* < 0.001). There has been no prospective randomised study comparing FOLFIRINOX to GN till date [23]. Retrospective studies comparing both have suggested greater activity for FOLFIRINOX, with a greater use of the FOLFIRINOX regimen in patients with better ECOG PS and general fitness. However, real-world evidence in the form of a metanalysis of 3,813 patients did not show a statistical OS or PFS benefit with FOLFIRINOX as opposed to GN as a first-line therapy in metastatic PDAC, underscoring the need for a prospective study to answer this question [24].

Other acceptable first-line therapy options especially in patients with ECOG PS 2 and an unfavourable co-morbidity profile are single agent gemcitabine, single agent 5-FU/capecitabine, doublet chemotherapy regimens like FOLFOX, gemcitabine plus capecitabine. Where available, S-1 monotherapy represents a reasonable alternative to gemcitabine monotherapy for patients who prefer the convenience of an oral regimen. Patients with advanced PDAC should be concurrently offered aggressive supportive treatment of cancer related symptoms like pain, etc.

Only about 40%–50% of the patients with advanced pancreatic cancer go on to receive second-line therapy [25]. A majority of trials and studies in this setting have been done in patients with good ECOG PS of 0–1, which is not the case in a real-world setting. Additionally, these were conducted in the pre-FOLFIRINOX era and evaluated oxaliplatin based chemotherapy after failure of gemcitabine which is applicable to only a very small subset of patients in the current era. Nanoliposomal irinotecan, a novel formulation of irinotecan has been evaluated in a phase III trial (NAPOLI-1) [26]. It showed that patients assigned to Nanoliposomal irinotecan plus 5-FU arm had a longer OS than patients treated with 5-FU alone (median, 6.1 versus 4.2 months; HR, 0.75; *p* = 0.012). Other efficacy endpoints like progression-free survival, objective response rates (RR) and time to treatment failure were also significantly superior in the Nanoliposomal irinotecan plus 5-FU arm. Two other studies with contrasting results with regard to the use of FOLFOX as a second-line regimen deserve mention in this context. The CONKO-003 trial showed a statistically significant OS benefit with FOLFOX in comparison with monotherapy with 5-FU/leucovorin (5-FU/LV) alone while the PANCREOX study did not show a benefit with using FOLFOX [27, 28]. Possible reasons for these differences, within the limitations of a cross trial comparison, included the lower doses of oxaliplatin and resulting better tolerance with FOLFOX in the CONKO-003 trial.

The data on second-line treatment options after prior FOLFIRINOX or GN and vice versa is limited to retrospective studies only. The major society guidelines suggest the choice of second line depending on the ECOG PS, co-morbidities and organ function. After first-line treatment with gemcitabine-based regimens, Nanoliposomal irinotecan plus 5-FU is the preferred option. 5-FU plus irinotecan or 5-FU plus oxaliplatin can be offered when Nanoliposomal irinotecan is not available. The choice of oxaliplatin or irinotecan depends on the pre-existing neuropa thy, though a metanalysis suggested a greater benefit for irinotecan containing combinations as compared to oxaliplatin containing combinations [29]. After first-line treatment with FOLFIRINOX, GN can be considered.

#### Treatment of frail and elderly patients

An aspect of the management of advanced PDAC that has been addressed to a very limited extent is the optimal management of patients who have an ECOG PS > 1 or patients who are not fit for intensive protocols like FOLFIRINOX. Additionally, this cohort of unaddressed patients increases when elderly patients with cancer are also considered. The median age at diagnosis of PDAC, as per the SEER database is approximately 70 years, yet a majority of clinical trials have very limited representation of these patients in their study populations [30]. For e.g., both the Unicancer GI PRODIGE trial evaluation FOLFIRINOX and the MPACT study evaluating nab-Paclitaxel had only 0.6% and 7% of patients with ECOG PS 2. Again, the median age of the patients in this study was 61 and 63 years, respectively. The proportion of patients aged greater than 65 was 22% and 42%, respectively [22, 23]. This is to emphasise the limited evidence available for the management of such patients in terms of clinical trials. In stark contrast, frail and elderly patients often comprise anywhere between 8% and 67% of patients in routine clinical practice and are treated with modifications of existing regimens with unclear evidence of benefit or lack, thereof [31, 32].

In the context of this frail population, available prospective evidence comes from two well conducted studies. The first is the seminal study by Burris *et al* [33], which showed a benefit for palliative chemotherapy. The study evaluated gemcitabine versus 5-FU in patients with advanced PDAC and showed a relative benefit for gemcitabine in terms of palliation, tolerance and survival. Sixty-nine percent of patients in this trial had an ECOG PS of ≥2, which is why gemcitabine is still a valid option in such patients [33]. The second study evaluating such a population was the FRAGRANCE trial, a phase I/II trial evaluating different dosing and scheduling of gemcitabine and nab-paclitaxel in patients with ECOG PS 2. The conclusions of the authors were that administering nab-Paclitaxel at either 100 and 125 mg/m^2^ in combination with gemcitabine on a weekly schedule of 3 weeks on, 1 week off, was well tolerated with reasonable safety and efficacy in frail patients presenting with ECOG PS 2 and advanced PDAC [34].

Treatment guidelines like NCCN and ESMO are relatively ambiguous on the management of such patients [18, 35]. The ESMO guidelines state, ‘In very selected patients with ECOG performance status 2 due to heavy tumour load, gemcitabine and nab-paclitaxel can be considered for best chance of response. If the performance status of the patient is 2 and/or the bilirubin level is higher than 1.5× ULN: a monotherapy with gemcitabine should be considered’. Tumour burden is an ill-defined characteristic that has predominant subjective characteristics as well as inter-observer variability and hence may not always be a valid benchmark for selection of treatment.

### Neoadjuvant therapy (NAT) in PDAC

The concept of neoadjuvant therapy (NAT) in a majority of solid tumours is based on the premise that it would increase margin negative resection rates, decrease extent of resections, provide an assessment of disease biology in terms of chemo-responsiveness and most importantly, potentially provide survival benefit. This has been proven and considered current standard practice in oesophageal, gastric and rectal cancers. However, with regard to PDAC, the biological evidence for the action of NAT has not been convincingly converted into clinically relevant data until recently. Multiple reasons exist for this. Firstly, while chemotherapy improved survivals post resection, RR with older regimens (predominantly gemcitabine, gemcitabine-platinum, 5-FU) were low in patients with advanced cancers [36]. This diluted the enthusiasm for using such regimens as NAT, though the advent of more efficacious regimens like FOLFIRINOX has overcame such lacunae in recent years [37–40]. Secondly, the negative impact of margin positive resections and significant morbidity and lack of survival benefit with extensive arterial resections has resulted in only a recent acceptance of classifying PDAC into resectable, borderline resectable pancreatic cancer (BRPC) and locally advanced PDAC (LAPC) [19, 21, 41–44]. Such a division has allowed multimodality management of BR/LA PDAC as the norm as opposed to concentration on surgical approaches alone. This approach has led to a near universal agreement on neoadjuvant strategies in BRPC with an increased intent for appropriate resections (40%–80%) while LA cancers are understood to require more conservative management with predominantly systemic therapy and resections only in a smaller proportion (1%–25%) [37, 45–47]. Thirdly, radiological responses post NAT, with chemotherapy or chemoradiotherapy, in PDAC, are mainly reflected by isovolumetric tissue replacement through fibrosis, rather than volume loss. A CT scan tends to overestimate residual tumour burden after NAT and does not accurately predict for the probability of R0 resection. This also leads to ambiguity in attempting resection post NAT [48]. Finally, most earlier studies have used long course radiotherapy (LCRT) along with gemcitabine as NAT for PDAC. Besides gemcitabine monotherapy being a weak agent against PDAC, there has been concern with regard to the development of micro-metastases during the course of LCRT as there is inadequate systemic coverage against a disease which has early systemic dissemination [49, 50]. These concerns have been mitigated to some extent by the development of techniques like stereotactic body radiation therapy (SBRT), though large-scale prospective evidence is lacking for their use.

While BRPC and to a certain extent, LAPC are treated with NAT in the current era with near universal acceptance, there are a number of studies which are also evaluating the role of NAT in resectable PDAC. Selected recent large studies using neoadjuvant chemoradiation (NACTRT) and/or neoadjuvant chemotherapy (NACT) with FOLFIRINOX and GN are discussed below.

#### NAT in BRPC and LAPC

In a large prospective cohort study of 680 patients with 574 assessable patients (BRPC – 39%, LAPC – 61%) by Maggino *et al* [51], the most commonly used NAT was FOLFIRINOX (53%), followed by GN (21%). Complementary radiotherapy (RT) was used in 25% of patients. Resection rates were 24% and 9% for BR and LA cohorts, respectively. The median survival for the whole cohort was 12.8 months (95% CI, 11.7–13.9 months). Factors independently associated with survival included completion of chemotherapy, application of RT and resection [51].

A systematic review and patient-level meta-analysis on neoadjuvant FOLFIRINOX in patients with BRPC comprising 24 studies and 313 patients showed the feasibility and effectiveness of such an approach. The resection rate noted was 67.8% with R0-resection rates of 83.9%. The mOS varied from 11.0 to 34.2 months across studies with a patient-level mOS of 22.2 months (95% CI, 18.8–25.6 months), and patient-level median PFS of 18 months (95% CI, 14.5–21.5 months) [38]. A similar patients-level metanalysis of 689 (315 LAPC with survival data) patients treated with FOLFIRINOX reported a mOS ranging from 10 to 32.7 months with a patient-level mOS of 24·2 months. The median PFS ranged from 3 to 20.4 months with a patient-level median PFS of 15.0 months. Surgical resection rates noted were 25.9% [37].

One of the earliest prospective studies using neoadjuvant FOLFIRINOX was a feasibility phase II study by Katz *et al* [52], wherein 22 patients with BRPC received FOLFIRINOX followed by concurrent capecitabine-RT. Fifteen of the 22 patients (68%) underwent pancreatectomy with 14 (93%) having R0 resections. The mOS of all patients was 21.7 months (95% CI, 15.7 to not reached) [52].

The phase II LAPACT study, comprising 107 patients, evaluated the GN regimen in patients with LAPC, and showed 58% completion rate of induction treatment, a disease control rate of 78%, median PFS of 10.9 months and mOS of 18.8 months. Sixteen percent of patients underwent resection [53].

A phase II/III trial from South Korea enrolled 58 patients with BRPC and evaluated NACTRT (54 Gy RT with concurrent gemcitabine) followed by surgery versus upfront surgery followed by adjuvant chemoradiation. In the intention-to-treat analysis, the median survival and R0 resection rates were significantly better in the NACTRT arm than the upfront surgery arm (21 months, 52% versus 12 months, 26%), though it is to be noted that the trial closed prior to its planned accrual of 110 patients, thereby impacting interpretation of the results [54].

One of the largest and most important prospective studies in the space of BRPC is the recently published Phase III PREOPANC trial. In this randomised phase III trial comprising 246 patients with BRPC or resectable PDAC, patients were randomly assigned to receive NACTRT (Gemcitabine for two courses and a third course concurrent with RT) followed by surgery and adjuvant gemcitabine or to immediate surgery and six courses of adjuvant gemcitabine. The initial results of the study showed that the primary endpoint of improved OS with NACTRT (16.0 versus 14.3 months; HR, 0.78; 95% CI, 0.58–1.05; *p* = 0.096) was not met, though NACTRT was associated with superior disease-free survival (DFS) and R0 resection rates [55]. The subgroup analysis showed that NACTRT predominantly benefited the BPRC subgroup as opposed to limited or no benefits in group of patients who had resectable disease upfront. While the initial results appeared promising in terms of feasibility of a neoadjuvant approach, the lack of an OS benefit was disappointing. However, the long-term results post a follow-up approaching nearly 5 years suggest a definite OS benefit for using NACTRT as opposed to upfront surgery in the cohort of patients. Three- and five-year OS was 27.7% and 20.5% after NACTRT versus 16.5% and 6.5% after upfront surgery (HR, 0.73; 95% CI, 0.56–0.96; *p* = 0.025). Other secondary outcomes such as DFS, locoregional failure-free interval and distant metastases free interval also improved with NACTRT [54]. The PREOPANC study has set the base for further studies evaluating the role of more efficacious regimens like FOLFIRINOX in the neoadjuvant setting to improve survivals in BRPCs as well as resectable cancers [57, 58].

Preclinical data has suggested that the renin-angiotensin system (RAS) activation in fibroblasts causes tumour fibrosis and desmoplasia, a key feature in PDAC. The primary effector for the RAS system is angiotensin-II, which is inhibited by Losartan via a receptor blocker mechanism. Using this principle, a single arm phase II trial combining FOLFIRINOX and Losartan with RT (either short course using protons or long course CTRT) in patients with LAPC showed impressive R0 resection rates of 69% and mOS of 31.4 months [59]. The investigators have initiated a further phase II study using the combination of FOLFIRINOX, Losartan, SBRT and Nivolumab in localised PDAC [60].

SBRT has emerged as a modality that overcomes the shortcomings of LCRT, while potentially increasing the chances of R0 resection margins when used as NAT [61]. However, the use of SBRT does not seem to improve 18-month survival rates as compared to historical controls in the Alliance A021501 trial. The phase II randomised non-comparative trial studied eight cycles of neoadjuvant modified 5-fluorouracil-leucovorin-irinotecan-oxaliplatin (mFOLFIRINOX) in one arm (Arm A) and seven cycles of mFOLFIRINOX followed by SBRT or hypo fractionated image guided RT] in the other arm (Arm B). The primary endpoint, 18-month OS rate, of each arm was compared to a historical control of 50%. The 18-month OS rate based on Kaplan–Meier estimates was 67.9% (95% CI, 54.6–78.0) in Arm A and 47.3% (95% CI, 33.7–59.7) in Arm B, with the ensuing interpretation that while neoadjuvant mFOLFIRINOX was associated with favourable OS relative to historical data, while mFOLFIRINOX with hypo fractionated RT did not compare favourably to historical data [62].

An update from the ESPAC-5F study, a prospective, international, randomised phase II trial of immediate surgery versus NACT or chemoradiotherapy has also provided some prospective evidence with regard to the benefit of NAT in BRPC. The study randomised 90 patients to either upfront surgery or NAT (either gemcitabine-capecitabine, FOLFIRINOX or concurrent capecitabine-RT). While there was no difference in R0 resection rates (surgery upfront – 44% versus NAT – 41%, *p* = 0.668), there was a significant OS difference at 12 months (surgery upfront – 42% versus NAT – 77%, *p* < 0.001) in favour of NAT. While the numbers are small, the maximum 12-month OS in any cohort was seen with neoadjuvant FOLFIRINOX (84%) [63].

#### NAT in resectable PDAC

The rationale for using NAT in BRPC/LAPC has also been used to push the case for NAT in resectable PDAC. The major concerns with using such an approach include delaying resection inordinately, thereby resulting in local progression of the tumour to unresectability, chemotherapy related complications resulting in inoperability, e.g., liver sinusoidal injury due to agents like oxaliplatin, and as previously mentioned, difficulties in assessing resectability by imaging post NAT [64, 65].

A number of studies using gemcitabine or gemcitabine-based combinations with or without RT have shown feasibility of NAT in resectable PDAC, though large-scale comparative prospective data is yet to emerge [66]. Besides the PREOPANC data, one of the largest studies evaluating NAT in resectable PDAC is the Prep-02/JSAP-05 trial from Japan. Patients received two cycles of gemcitabine plus S1 followed by resection and adjuvant S1 in one arm while in the other arm, patients underwent upfront resection followed by adjuvant S1. The study enrolled 364 patients and achieved its primary endpoint of improved OS in the NAT arm (mOS, 36.7 versus 26.6 months; HR, 0.72; 95% CI, 0.55–0.94; *p* = 0.015). There were no differences in the resection rate, R0 resection rate and perioperative morbidity, though an increased incidence of neutropenia was noted in the NAT arm [67].

A smaller prospective ‘pick the winner design’ phase II randomised study, looking at outcomes with either FOLFIRINOX (Arm 1) or GN (Arm 2) as compared to historical cohorts, did not suggest an improvement in OS in patients with resectable PDAC. In the 102 evaluable patients, 73% in Arm A and 70% in Arm B underwent resection. Two-year OS was 47% (95% CI, 31%–61%) for Arm 1 and 48% (95% CI, 31%–63%) for Arm 2. As per the authors, neither arm’s 2-year OS estimate was significantly higher than the *a priori* threshold of 40% [68]. The study is important in the context of highlighting the need for precise radiological assessment of resectability in PDAC as well as the fact that there were no significant differences between the two arms of the study in terms of tolerance and efficacy when used as NAT. However, the lack of a significant improvement in 2-year OS highlights the need for further clarification on the role of NAT in resectable PDAC. Table 3 highlights key features of select studies which have evaluated NAT in PDAC.

A systematic review and metanalysis comprising eight studies and approximately 1,300 patients (predominantly BRPC/LAPC, though with a cohort of resectable PDAC) receiving either FOLFIRINOX or GN suggested that FOLFIRINOX showed improved 1-, 2- and 3-year survival rates compared to GN. However, R0 resection rates and 4- and 5-year survival rates showed similar results [69].

### Surgical challenges in pancreatic resections post NAT

#### Assessment of tumour response for resection post NAT

Contrast enhanced CT scan remains the primary tool in initial categorisation of pancreatic cancer into resectable, BRPC or LAPC. However, as previously described, CT imaging frequently underestimates tumour response post NAT. It fails to discriminate between viable tumour and fibrotic tissue [70]. ‘Halo sign’ and ‘string sign’, respectively, suggest actual vessel wall involvement or periadventitial involvement of superior mesenteric artery (SMA) by the tumour which may help in assessing operability. Functional imaging via PET scans and changes in CA 19.9 may also help guide management decisions. Though there is no definite recommendation, in absence of disease progression on reassessment imaging all patients of pancreatic cancer should be explored for surgery post NAT [48].

### Options of systemic therapy beyond chemotherapy

Precision medicine has led to the identification of specific targets in various cancers and has led to some success in the management of advanced cancers. As previously described, targets have been identified in PDAC as well, though disappointingly, very few ‘druggable’ targets have achieved relevance in clinical trials and practice. Commonly used monoclonal antibodies that are active in other cancers and target EGFR (e.g. cetuximab, erlotinib, etc.) and VEGF (e.g. bevacizumab), besides others, have shown disappointing results in PDAC [71–74].

Olaparib, a Poly (ADP-Ribose) Polymerase inhibitor, targets cancer cells with a homologous recombination repair deficiency, such as due to BRCA gene mutation, by synthetic lethality. Germline loss-of-function mutations in BRCA1, BRCA2 or both (BRCA) genes occur in approximately 4%–7% of patients with PDAC. The phase III Pancreas Cancer Olaparib Ongoing trial randomised patients with advanced PDAC post 16 weeks of continuous first-line platinum-based chemotherapy to receive either olaparib (600 mg/day) or placebo as a form of maintenance therapy. The study achieved its primary endpoint of showing improved PFS (7.4 versus 3.8 months; HR, 0.53; 95% CI, 0.35–0.82; *p* = 0.004) with olaparib and is the first instance of a targeted therapy showing benefit in advanced PDAC [75].

KRAS is the most common genetic alteration in PDAC and has long been considered as ‘non-druggable’. However, two molecules (AMG 510 and MRTX849) which use covalent allosteric inhibition to target a shallow pocket on the KRAS surface, specifically against the KRASG12C codon, have shown promising activity in early phase studies. Approximately 1%–4% of PDACs have KRASG12C codon mutation and these drugs may be considered in the future for treatment [76].

Gene fusions involving neurotrophic tyrosine receptor kinases (NTRKs) have been identified in approximately 1% of solid tumours and inhibitors of these kinases have been shown to have activity in a tumour agnostic manner [77, 78]. These fusions appear to be even rarer in PDAC, though case reports showing activity for NTRK inhibitors in advanced PDAC have been noted [79, 80]. Immune checkpoint inhibition by using antibodies against cytotoxic T-lymphocyte-associated antigen 4, programmed cell death protein-1 and programmed cell death protein ligand-1 has revolutionised the management of non-small cell lung carcinoma, melanoma, urothelial cancers, renal cell carcinomas amongst other cancers [81]. The strongest biomarker for the use of immune checkpoint inhibitors is a deficient mismatch repair (dMMR) protein status. The MMR proteins are key in error repair during DNA replication, with a defective system leading to random mutations occurring in small repetitive elements called microsatellites, i.e. microsatellite instability (MSI). Available evidence shows that a dMMR (MSI-H) status is rarely seen in PDAC, approximating a prevalence of 1%–2% [82]. While this is rare, immune checkpoint inhibitors may be considered in patients with PDAC and a dMMR status, though the limited available evidence suggests lesser responses with immunotherapy in PDACs as compared to responses seen in other tumours [83]. Other forms of immunotherapy like vaccines and adoptive cell transfer have not proven to be successful in PDAC, though there is emerging data for combination therapy, e.g. chemotherapy and pembrolizumab, ipilimumab plus GVAX vaccine, etc. [5].

#### Vascular resections and ‘Artery first approach’

With the advent of effective NAT regimens (FOLFIRINOX) and surgeons advancing their limits of resection, a proportion of initially unresectable (UR) patients now undergo successful resection.

Survival and perioperative outcomes of patients undergoing vein resection during pancreatectomy are similar to patients undergoing standard pancreatectomy. Porto-mesenteric vein resection has been accepted as a standard of care in surgery for BRPC [70, 84]. Tumour encasement resulting in complete collateralisation and cavernous transformation of porto-mesenteric vein complex and absence of reconstructible venous stumps are the only venous criteria for inoperability [85].

Except for celiac artery (CA) involvement in pancreatic body cancers and short segment common hepatic or gastroduodenal artery involvement in case of right-sided pancreatic cancers, arterial resection is considered a contraindication for surgery. This recommendation stems from the experience of high morbidity, mortality in excess of 10% and limited survival benefit with major arterial resection, in most historical series [86, 87].

Unless an arterial resection is planned, early intraoperative assessment of tumour-artery interphase is important before committing irreversible steps of resection while exploring patients with BRPC or LAPC for possible resection. SMA first approach involves systematic dissection of pancreas off SMA to achieve negative margins of resection and assess operability before performing other major steps of resection. It allows surgeon to abandon the procedure in cases where complete resection seems unlikely. In addition to early assessment of operability, this approach has been shown to improve perioperative outcomes such as blood loss, fistula rate and delayed gastric emptying and long-term survival [88–90].

#### Arterial Divestment, Periadventitial dissection and triangle operation

To minimise morbidity and to avoid arterial resection when disease does not directly involve the artery, periadventitial SMA dissection technique (also called as arterial divestment) has been described. This dissection technique also labelled as ‘level 3’ mesopancreas dissection, by Inoue *et al* [91], involves en block mesopancreas resection with right hemi or complete circumferential SMA dissection in periadventitial layer and complete clearance of tumour, lymphatics and perineural tissue around SMA. Such a procedure offers the possibility of achieving radical resection in LAPCs post NAT without arterial resection, thereby avoiding the morbidity and mortality associated with arterial resections. Long-term outcomes and further prospective evidence for such procedures should be the aim of future surgical studies [92].

### Adjuvant therapy in PDAC

Approximately 10%–15% of patients with PDAC are resectable at presentation. The important determinants of outcomes in resectable PDAC are the presence or absence of involved lymph nodes, resection margins status (beyond scope of current review) and administration of effective adjuvant chemotherapy (AT) [93, 94]. Lymph nodal involvement, number of lymph nodes involved as well as parameters like lymph node ratios have been repeatedly shown to be prognostic in major staging systems as well prospective studies evaluating adjuvant therapy [93, 95]. The major reason for the development of AT in resected PDAC was the early recurrence and dismal OS (approximately 10–12 months) post a major procedure like classical Whipple resection or pylorus preserving pancreaticoduodenectomy [96]. The first study to show randomised evidence for the benefit of AT was the Gastrointestinal Tumor Study Group study published in 1985. The investigators found a near doubling of OS (21 versus 10.9 months; *p* = 0.03) when combining 5-FU with RT post resection as opposed to surveillance alone [97]. Since the advent of this trial, a number of prospective trials have shown the importance of AT in improving survival. Studies over the last two decades have concentrated on intensifying adjuvant chemotherapeutic regimens, while adjuvant RT has fallen out of favour due to lack of survival benefit and issues with tolerance [98–101]. The current standard of care for patients with resected PDAC is adjuvant FOLFIRINOX biweekly for 6 months. This is based on a Phase III randomised control trial, which compared modified FOLFIRINOX with gemcitabine monotherapy in 493 patients and showed a statistically significant improvement in the primary endpoint of DFS (21.6 versus 12.8 months; stratified HR, 0.58; 95% CI, 0.46–0.73; *p* < 0.001). A significant difference in OS was also noted in favour of the modified FOLFIRINOX arm (54.4 versus 35 months; stratified HR, 0.64; 95% CI, 0.48–0.86; *p* = 0.003) [102]. While the modified FOLFIRINOX regimen has shown the best survivals in this setting, the regimen also entails a significant proportion of patients having grade 3 or grade 4 adverse events (75.9%). This coupled with delayed recovery and nutritional depletion post pancreatic resections means a significant proportion of patients may be unable to receive mFOLFIRINOX. In such a cohort of patients, regimens for which evidence exists include combination gemcitabine-capecitabine, S1 or gemcitabine monotherapy [100, 103, 104]. Table 4 lists the major large randomised adjuvant therapy trials and the significant results associated with these trials.

### PDAC – Data from India

#### Epidemiological data

As per the GLOBOCAN 2020 India factsheet, PDAC ranks 24th in terms of incidence with 12,642 new cases (0.95%) and 19th in terms of mortality with 12,153 cases (1.4%) [1]. The data published from 28 population-based cancer registries (PBCRs) of India, puts the cumulative risk of pancreatic cancer for males and females at 1 in 429 and 1 in 519, respectively, for the year 2020 [105]. The north-eastern region of India, especially Mizoram, has higher incidence rates as compared to other regions of the country. The age-adjusted incidence rates of PDAC have increased by more than two-fold as per the data from four PBCR in India over a period spanning from 1982 to 2010 [106]. The peak incidence rates are a decade earlier in India (sixth decade) as compared to that of the United States (seventh decade) [107].

#### Practice patterns and survival

The practice of pancreatic surgeries and perioperative management has evolved over the last few decades in India, just like in the developed countries. This has led to a significant decrease in the post-operative surgical complications. In one of the largest series on perioperative outcomes for pancreaticoduodenectomy from a tertiary care centre in India, the reported rates of morbidity and mortality were 33% and 5.4%, respectively [108]. The authors from this series reported a significant drop in the overall morbidity rates for surgeries done post 2003 as compared to the ones done a decade earlier. There was no statistically significant difference in the mortality rates, though. Such improvements in volume and safety indicators were noted despite the significant increase in the volume of cases per year (from 16 to 60; *p* < 0.0001). The 2-year DFS rates for those who underwent surgery post 2003 were 75% in this series.

Limited retrospective studies have suggested that patients with advanced PDAC in India have similar outcomes compared to published data [3, 46]. The systemic treatment for UR locally advanced and metastatic pancreatic cancer has also evolved from single agent gemcitabine to combination regimens like GN, and mFOLFIRINOX, thereby improving the survival and outcomes. A retrospective analysis of metastatic pancreatic cancer patients treated at a tertiary centre between 2013 and 2016 showed the most commonly used first-line regimens to be GN (39.2%), gemcitabine–erlotinib (16.3%) and mFOLFIRINOX (13.7%) [3]. The proportion of patients receiving mFOLFIRINOX (6.9% to 13.7%) and GN (5.9% to 39.2%) had increased, when compared to earlier published data from the same institution. Nearly 44% of patients received chemotherapy with dose modifications, out of which more than a half were upfront dose-modifications due to poor nutrition, co-morbidities and ECOG PS 2. With a median follow-up of approximately 9 months, the reported mOS was 7 months [3, 109]. A prospective study (published in abstract form only till date) comparing FOLFIRINOX with gemcitabine showed improved OS with FOLFIRINOX, at the cost of an increased toxicity profile. The OS in both arms of the study was in line with the data from the 4-ACCORD11 trial [110]

Approximately 40% of patients with inoperable pancreatic cancer receive second-line chemotherapy [111]. Similar rates have been reported from Indian literature too, with the most common regimens used being modified FOLFIRI (used without bolus 5FU) and GN [112]. The choice of regimen in later lines depends heavily on the ECOG PS and the regimen used prior. With a median follow-up of 7.57 months, a retrospective series reported an mOS of approximately 8 months.

## Conclusions

A greater understanding of the biology of PDAC has resulted in a gradual shift towards intensifying systemic management options in PDAC, with accompanying standardisation of surgical approaches. The recalcitrant nature of PDAC is intrinsically related to the lack of therapeutic targets and dense surrounding stroma that hampers effectiveness of currently available chemotherapeutic options. An additional issue is the presentation of these cancers in predominantly advanced stages of disease. Despite these constraints, gradual improvements in survival are being seen with more effective chemotherapeutic regimens like mFOLFIRINOX and GN, whether in resectable or advanced PDAC. An increasing use of these effective chemotherapeutic regimens has also resulted in greater conversion of BR and locally advanced cancers to resection, though the most effective approach in this subgroup is yet to be identified. Available data from India shows similar outcomes across the spectrum of PDAC when compared to published data.

## Funding sources and grants

None.

## Conflicts of interest

None.

## Figures and Tables

**Table 1.** Resectability criteria in PDAC (NCCN criteria) [19].

Resectability status	Arterial	Venous
Resectable	SMA, CA, CHA: no arterial tumor contact	SMV/PV: no tumor contact, or contact of less than 180° without vein contour irregularity.
Borderline resectable (BR)		
BR- PV (SMV/PV invasion alone)	-	SMV/PV: solid tumor contact of 180° or more, contact of less than 180° with contour irregularity of the vein or thrombosis of the vein but with suitable vessel proximal and distal to the site of involvement allowing for safe and complete resection and vein reconstruction. IVC: solid tumor contact
BR-A (arterial invasion)	*Pancreatic head/uncinate process*: SMA: solid tumor contact of less than 180° CHA: solid tumor contact without extension to CA/hepatic artery bifurcation allowing for safe and complete resection and reconstruction. Presence of variant arterial anatomy (RHA, CHA) and the presence of tumor contact as it may affect surgical planning *Pancreatic body/tail:* CA: solid tumor contact of less than 180° CA: solid tumor contact of 180 or more degree without involvement of the aorta and with intact and uninvolved GDA	-
Unresectable (UR)		
UR-LR (locally advanced)	*Head/uncinate process*: SMA, CA: solid tumor contact of 180 or more degree Solid tumor contact with the 1st jejunal SMA branch *Body and tail* SMA, CA: solid tumor contact of 180 or more degree Solid tumor contact with the CA and aortic involvement	*Head/uncinate process*: SMV/PV: unreconstructible due to tumor involvement/occlusion Contact with most proximal draining jejunal branch into SMV *Body and tail* SMV/PV: unreconstructible due to tumor involvement/occlusion
UR-M (metastatic)	Distant metastasis (including non-regional lymph node metastasis)	

SMV: Superior mesenteric vein, PV: Portal vein, SMA: Superior mesenteric artery, CA: Celiac artery, CHA: Common hepatic artery, IVC: Inferior Vena Cava, RHA: Right Hepatic Artery, GDA: Gastroduodenal Artery, LR: Locally advanced

**Table 2.** Selected trials on first-line treatment in advanced pancreatic cancers.

Trial	Types of trial	*N*	Intervention	Primary endpoint	RR (%)	mDFS (months)	mOS (months)	Result
Burris III et al [33]	RCT phase III	126	G vs. bolus 5-FU	‘Clinical benefit’	5.4 vs 1	2.33 vs 0.92	5.65 vs 4.4	Achieved primary end point
National Cancer Institute of Canada Clinical Trials Group [71]	RCT phase III	569	G plus erlotinib vs. gemcitabine	OS	8.6 vs 8	3.75 vs 3.55	6.24 vs 5.91	Achieved primary end point, though limited clinical relevance
PRODIGE 4-ACCORD 11 [22]	RCT phase III	342	FOLFIRINOX vs. G	OS	31.6 vs 9.4	6.4 vs 3.3	11.1 vs 6.8	Achieved primary end point
MPACT [23]	RCT phase III	861	G- nab-paclitaxel vs. gemcitabine	OS	23 vs 7	5.5 vs 3.7	8.5 vs 6.7	Achieved primary end point

*N*, Total no of patients; RR, Response rates; mDFS, Median disease-free survival; mOS, Median overall survival; RCT, Randomized clinical trial; OS, Overall survival; G, Gemcitabine; 5 FU, 5 Fluorouracil; FOLFIRINOX, 5-fluorouracil-leucovorin-irinotecan-oxaliplatin

**Table 3.** Selected studies evaluating neoadjuvant treatment (NAT) in PDAC.

Study/first author	Types of study	*N*	Interventions	Resection rates	mOS (months)	Interpretation
**BRPC/LAPC**						
Sadot et al [39]	R	0/101	FOLFIRINOX +/- CTRT	31	25	Feasibility
Rangelova et al [40]	R	22/132	FOLFIRINOX	33(BRPC/NR (LAPC)	31.9(BR)/21.8 (LA)	Feasibility
Maggino et al [51]	R	267/413	53% FOLFIRINOX 21% G-Nab-Paclitaxel 25% RT	15.1	12.8	Feasibility
Katz et al [52]	Single arm, phase II	22/0	FOLFIRINOX + CTRT	68	22	Feasibility
Hammel et al [61]	RCT, Phase III	0/442	G-Erlotinib vs. G-Erlotinib + CTRT	4	16.5 vs. 15.2	CTRT did not improve OS
Janssen et al [38]	M	313/0	FOLFIRINOX	67.8	22.2	Patient level metanalysis
Suker et al [37]	M	0/315	FOLFIRINOX	25.9	24.2	Patient level metanalysis
Jang et al [54]	RCT, Phase II/III	58/0	NACTRT → Sx vs. upfront Sx	52 vs. 26	21 vs. 12	Improved resection rates and OS with NACTRT
PREOPANC [55,56]	RCT, Phase III	246 (BRPC and Resectable)	NACTRT → G --> Sx vs. Sx → G	61 vs. 72	16 vs 14.3	Neoadjuvant approach did not improve OS
Alliance A021501 [62]	RCT, Phase II	126	FOLFIRINOX → Sx vs. FOLFIRINOX + SBRT → Sx	51 vs 58	31 vs. 17.1	Addition of SBRT did not improve resectability rates compared to historical controls
**Resectable PDAC**						
O’ Reilly [66]	Phase II, single arm	38	GEMOX → Sx	71	27.2	Feasibility
Prep-02/JSAP-05 [67]	RCT, Phase II/III	364	G-S1 → Sx → S1 vs. Sx→ S1	Similar	36.7 vs. 26.6	NAT with G-S1 improved OS
SWOG S1505 [68]	Phase II, Pick the winner design	102	FOLFIRINOX → Sx vs GN → Sx	73 vs 70	23.2 and 23.6	Both arms did not improve OS compared to historical cohorts

*N*, Total no of patients; mOS, Median overall survival; Sx, Surgery; RCT, Randomized clinical trial; OS, Overall survival; G, Gemcitabine; 5 FU, 5 Fluorouracil; FOLFIRINOX, 5-fluorouracil-leucovorin-irinotecan-oxaliplatin; GEMOX, Gemcitabine-oxaliplatin; RT, Radiotherapy; NACTRT, Neoadjuvant chemoradiation; NACT, Neoadjuvant chemotherapy; NR, Not reported; SWOG, South West Oncology Group

**Table 4.** Randomised large-scale trials for adjuvant therapy of resected PDAC in the modern era.

Trial	Types of trial	*N*	Interventions	Primary endpoint	mDFS (months)	mOS (months)	Result
CONKO-001 [98]	Phase III	368	G. vs. Observation	DFS	13.4 vs. 6.7	22.8 vs. 20.2	Achieved primary endpoint
RTOG 9704 [99]	RCT, Phase III	451(Pancreatic-388)	5-FU/Lv, → concurrent 5-FU-RT, --> 5-FU/Lv; vs. G.--> concurrent 5-FU-RT, followed by G.	OS	NR	20.5 vs. 17.1	Trend towards benefit with G, but statistically did not achieve endpoint
ESPAC-3 [101]	RCT. Phase III	1088	G. vs. 5 -FU	OS	14.3 vs. 14.1	23.6 vs. 23	Did not achieve primary endpoint
JASPAC-01 [100]	RCT, Phase III (Non inferiority)	385	G. vs. S1	OS	11.3 vs. 22.9 (RFS)	25.5 vs. 46.5	Achieved primary endpoint
ESPAC-4 [103]	RCT, Phase III	732	G-C. vs. G	OS	13.9 vs. 13.1	28 vs. 25.5	Achieved primary endpoint
Canadian Cancer Trials Group and the Unicancer-GI–PRODIGE Group [102]	RCT, Phase III	493	mFOLFIRINOX vs. G.	DFS	21.6 vs. 12.8	54.4 vs. 35	Achieved primary endpoint
APACT [104]	RCT, Phase III	866	nab-P/G vs. G.	DFS	19.4 vs. 18.8	40.5 vs. 36.	Did not achieve primary endpoint

*N*, Total number of patients; mDFS, Median Disease-free survival, mOS, Median Overall survival, JASPAC, Japan Adjuvant Study Group of Pancreatic Cancer; RTOG, Radiation Therapy Oncology Group**,** APACT, Adjuvant Therapy for Patients With Resected Pancreatic Cancer**;** G, Gemcitabine, 5-FU/LV - 5-fluorouracil/leucovorin, RT, Radiotherapy, RFS, Recurrence free survival, G-C, Gemcitabine-capecitabine, mFOLFIRINOX, Modified 5-fluorouracil-leucovorin-irinotecan-oxaliplatin, nab-P/G, Nab-Paclitaxel/gemcitabine, NR, Not reported.
